# The first chromosome-scale genome assembly for *Dermacentor reticulatus*: a key vector of tick-borne pathogens of public and veterinary health importance in Europe

**DOI:** 10.1186/s12915-026-02646-z

**Published:** 2026-06-11

**Authors:** Nina Billows, Mia L. White, Joseph Thorpe, Matthew Higgins, Emma Collins, Myrela Santos de Jesus, Mojca Kristan, Steven Pullan, Sarah M. Biddlecombe, Faye V. Brown, Taane G. Clark, Kayleigh M. Hansford, Jolyon M. Medlock, Susana Campino

**Affiliations:** 1 Faculty of Infectious and Tropical Diseases, London School of Hygiene and Tropical Medicine, London, UK; 2 Diagnostics & Pathogen Characterisation, UKHSA, Porton Down, Salisbury, SP4 0JG UK; 3 Faculty of Epidemiology and Population Health, London School of Hygiene and Tropical Medicine, London, UK; 4 Medical Entomology and Zoonoses Ecology, UKHSA, Porton Down, Salisbury, SP4 0JG UK

**Keywords:** *Dermancentor reticulatus*, Europe, Ticks, Genomics, Genome assembly

## Abstract

**Background:**

*Dermacentor reticulatus* is a key tick species across Europe and an established vector of multiple pathogens affecting both human and animal health. Despite its significant role in disease transmission and its expanding distribution, genomic data for this species remain limited. Here, we present the first chromosome-scale genome assembly of *D. reticulatus*, constructed using Oxford Nanopore long-read sequencing.

**Results:**

This chromosome-scale genome assembly revealed a repeat-rich genome, with approximately 63.6% of the total sequence consisting of repetitive elements. BUSCO analysis demonstrated strong genome completeness, with guided assembly achieving a score of 97.9%, comparable to related *Dermacentor* species. Gene annotation predicted 21,592 genes with a BUSCO completeness score of 98%. Functional characterisation, including Pfam domain assignment and gene ontology analysis, highlighted enrichment of molecular functions associated with detoxification, protease regulation, antimicrobial defence, and cholesterol metabolism. Additionally, the mitochondrial genome (15,103 bp), comprising 38 genes, was assembled, providing further insight into *D. reticulatus* phylogenetic placement in the *Dermacentor* genus.

**Conclusions:**

This genomic resource establishes a foundation for studying tick biology, evolution, and host–pathogen interactions.

**Supplementary Information:**

The online version contains supplementary material available at 10.1186/s12915-026-02646-z.

## Background

*Dermacentor reticulatus*, also known as the ornate cow tick or European meadow tick, is a vector of medical and veterinary importance, capable of transmitting a broad range of pathogens. Predominantly found across Europe and parts of Western Asia, *D. reticulatus* thrives in a variety of habitats with preference for grasslands, pastures, meadows, sand dunes, marshy areas, and grassy paths in woodlands. This three-host species commonly feeds on mammals, such as rabbits, deer, horses, and livestock, while also opportunistically biting humans and companion animals, especially dogs [1–4]. In the UK, *D. reticulatus* has mostly been observed across coastal regions of Southwest England and West Wales associated with sand dune rabbit burrow systems and coastal maritime grassland, as well as urban green spaces in Essex [5]. Historically, the distribution of *D. reticulatus* was confined to regions with milder climates [6]. However, this species has expanded into cooler and more northerly regions, including Belgium, the Netherlands, Germany, Poland, Hungary, Slovakia, and new foci in South East England [3, 5, 6] and is essentially known as a winter tick.

This trend is mostly associated with movement of infested livestock but could be driven by other anthropogenic factors [5, 7, 8]. The wide geographic distribution of *D. reticulatus* raises public and animal health concerns, as this tick is a known vector for some pathogens (e.g. *Babesia*, *Anaplasma*, *Rickettsia*, *Theileria*) and has been found to carry other agents, including *Bartonella*, *Francisella*, *Coxiella*, Omsk haemorrhagic fever virus, and tick-borne encephalitis virus, though vector competence for these remains unconfirmed [9, 10]*.* Surveillance of *D. reticulatus* is therefore essential for early detection of range shifts and associated disease threats. While the morphology and some elements of the ecology of *D. reticulatus* are well characterised, including its high reproductive rate, flexible habitat preferences, and ability to transmit diverse pathogens, there remains a significant genomic knowledge gap [10]. This gap limits molecular-level understanding of its biology and vector capacity.

Currently, no chromosome-scale reference genome exists for *D. reticulatus*. Genomic resources are critical for accurate species identification, detection of cryptic population structure, and monitoring of pathogen carriage. Moreover, they enable population genomics and comparative analyses to uncover adaptive traits linked to vector competence, climate tolerance, or ecological plasticity. The use of microsatellite loci and mitochondrial sequence data has provided some insight into *D. reticulatus* ancestral admixture [11]. However, further whole genome sequencing studies are invaluable for enhancing tick-borne disease surveillance and informing the development of novel vector control strategies, with the potential for reducing the health risks posed by this expanding vector.

To date, reference genomes with varying quality have been generated for related *Dermacentor* species largely distributed across Asia and North America [11, 12]. For instance, *D. silvarum* (GCA_013339745.2), a dominant tick species in Northeast China, was sequenced using an integrated approach combining Illumina, PacBio, and Hi-C technologies, enabling chromosome-level resolution [12]. Three additional ticks belonging to the *Dermacentor* genus have chromosome-scale genome assemblies available, including *D. albipictus* (GCA_038994185.2), *D. andersoni* (GCA_023375885.3), and *D. variabilis*, all found within North America and Canada, while a scaffold-level is available for *D. reticulatus* (GCA_051549955.1) [13–16]. These genomic data provide a robust framework for comparative studies, but do not adequately represent European tick populations.

In response to the expanding distribution of *D. reticulatus* and the rising incidence of tick-borne diseases in Europe, we identified an urgent need for a reference genome for this species [10]. In this study, we present the first chromosome-scale genome assembly for *D. reticulatus*, generated from a female tick collected in Devon, England (March 2024), using Oxford Nanopore Technology (ONT) long-read sequencing. We evaluate assembly and annotation quality, conduct comparative analyses with other *Dermacentor* genomes, and highlight the genomic architecture and functional gene content associated with its hematophagous lifestyle. This assembly provides a foundational genomic resource to support future surveillance, population genomics, and functional studies aimed at better understanding the biology, adaptation, and control of this medically and veterinary important tick species.

## Methods

### Sample collection and sequencing

A single *D. reticulatus* female sample was collected in a known established area in Devon, England, using flagging. The sample was homogenised using a pestle and DNA was extracted using Dynabeads™ SILANE Genomic DNA Kit using the manufacturer’s instructions, to produce high genomic weight DNA. The concentration of the DNA was assessed using the Qubit Fluorometer with the dsDNA high sensitivity kit (Invitrogen). The 4200 TapeStation system, with Genomic DNA ScreenTape assay (Agilent), was used to assess the quality and quantity of DNA, and the sample with the highest molecular weight DNA was selected (80% DNA with peak at 20,000 bp).

A DNA library of the *D. reticulatus* was prepared using the Ligation Sequencing Kit V14 (SQK-LSK114; Oxford Nanopore Technologies (ONT), UK), following the manufacturer’s protocol. The DNA library was sequenced on an R10.4.1 PromethION flow cell (FLO-PRO114M; ONT, UK). Raw reads were basecalled using Dorado v0.9.1 (super high accuracy model) to convert the sequence signals (‘squiggle’) with –trim to remove adaptor sequences.

RNA was extracted from a single homogenised whole adult tick using the RNeasy Mini Kit (Qiagen), following the manufacturer’s protocol. Ribosomal RNA was subsequently depleted and sequencing libraries were prepared and sequenced by Novogene on an Illumina platform to generate paired-end reads. Raw reads were quality filtered and adapter trimmed using fastp v0.23.4 [17]. Reads were subsequently mapped to the *D. reticulatus* reference genome (see “Genome assembly”) using HISAT2 v2.2.2 to remove potential contaminants prior to downstream analyses. This yielded 33.14 million read pairs for downstream annotation.

### Genome assembly

Kraken2 v2.1.3 was used to assign taxonomic labels to raw ONT reads and generate a contamination report [18]. No eukaryotic contaminants were reported and raw reads were subsequently filtered to remove sequences matching ‘Archaea’ in the Kraken2 report using KrakenTools (extract_kraken_reads.py) [19]. The remaining reads were scrubbed using yacrd v1.0.0 to exclude any poor quality or chimeric sequences [20]. Reads were filtered further using chopper v0.6.0 to extract reads with a minimum length of 1000 and minimum quality of 12 [21]. K-mer frequency analysis was performed using Jellyfish v2.2.10 and modelled with GenomeScope 2.0 (k-mer size = 27, ploidy = 2) to estimate genome size and heterozygosity [22, 23]. The high-quality reads underwent unguided reference assembly using Hifiasm 0.25.0-r726 (options –ont) [24]. Because the focus of this study was a single representative genome sequence rather than phased haplotypes, only the primary (combined) assembly produced by Hifiasm was used for downstream analyses. The unguided scaffolds underwent additional guided scaffolding using the available *D. silvarum* genome (GCA_013339745.2) using RagTag v2.1.0 [12, 25]. This genome was chosen as the reference guide due to its high-quality, close phylogenetic relationship with *D. reticulatus* as determined by mtDNA analysis (compared with other *Dermacentor* species), and expected chromosome number (previously determined by karyotyping: 10 autosomal chromosomes, 1 sex chromosome) [26]. The remaining gaps were filled using TGS-GapCloser v1.2.1 [25, 27]. Finally, the unguided and guided genome assemblies were polished using racon v1.5.0, before screening for contaminants and adaptors using the NCBI Foreign Contamination Screen (FCS) suite [28, 29]. The quality-filtered reads were mapped to the final reference sequence and coverage was assessed using mosdepth v0.3.10 in 1 kb windows [30]. All raw sequencing data and genome assemblies have been deposited at NCBI and are also available through the LSHTM Genomics Data Repository [31, 32].

### Genome assembly quality assessment

The quality of genome assemblies was assessed using BUSCO v5.8.2 (-m genome -l arachnida_odb10) after each stage of the pipeline to assess the impact of each process on quality [33]. Final quality assessments were conducted for the unguided and guided *D. reticulatus* genome assemblies and benchmarked against assembly metrics from other available *Dermacentor* genomes in the GenBank database, including *D. silvarum* (GCA_013339745.2, 11 chromosomes), *D. albipictus* (GCA_038994185.2, 10 chromosomes), and *D. andersoni* (GCA_023375885.3, 11 chromosomes) [12].

### Repeat masking and genome annotation

RepeatMasker v4.1.7 and the ‘Chelicerata’ Dfam database were first used to identify and classify repeats in the *D. reticulatus* genome [34–36]. This yielded a small percentage of repeats, prompting further de novo modelling of repeats using RepeatModeler v2.0.1 [37]. The results from both outputs were combined to hard and soft-mask sequences using RepeatMasker. Genome annotation was performed using the EGAPx v0.5.0 pipeline and *Dermacentor reticulatus* RNA short read data (https://github.com/ncbi/egapx). Additionally, tRNA sequences were also annotated using tRNAscan-SE. The quality of genome annotation was assessed using BUSCO v5.8.2 (-m protein -l arachnida_odb10) and was compared between the unguided and guided *D. reticulatus* assemblies and aforementioned *Dermacentor* sp. assemblies after removing alternative transcripts [33].

### Nucleotide and gene synteny

To further investigate potential misassemblies in the *D. reticulatus* assemblies, nucleotide-level synteny analysis was performed against the *D. silvarum* genome. Whole genome alignment was performed using minimap2 v2.28 (-ax asm5) between the *D. silvarum* reference and masked *D. reticulatus* assemblies [38]. Mapped regions were filtered to retain high-quality matches (MAPQ > 60) to exclude any potential unmasked repeats and were used to create a ‘links’ file. The links between the *D. reticulatus* assembly and *D. silvarum* were visualised using the PyCircos v3.10.0 package to interpret nucleotide-level synteny (https://github.com/ponnhide/pyCircos). In addition, synteny was also explored at the gene level. Coding sequences for *D. reticulatus* and *Dermacentor* sp. assemblies were extracted and inputted into jcvi and MCScan (Python version) for pairwise synteny searches and macrosynteny visualisation [39].

### Phylogenetic orthology inference

Analysis of orthologues was conducted using the OrthoFinder platform v2.5.5 [40]. Amino acid sequence files (*.faa) for the unguided and guided *D. reticulatus* assemblies were compared to other scaffold- or chromosome-level tick assemblies, including *D. albipictus* (GCF_038994185.2), *D. andersoni* (GCA_023375885.3), *D silvarum* (GCF_013339745.2), *Haemaphysalis longicornis* (GCA_013339765.2), *Ixodes scapularis* (GCF_016920785.2), *Ixodes persulcatus* (GCA_013358835.2), *Alectorobius turicata* (GCF_037126465.1), *Rhipicephalus microplus* (GCF_013339725.1), and *Rhipicephalus sanguineus* (GCF_013339695.2) [12, 15, 16, 41]. A tree based on orthogroups was built using the platform (-M msa [Default = mafft], -T iqtree). The resulting comparative genomics statistics were used for downstream analyses, and the tree was visualised using custom scripts.

### Functional annotation of protein sequences

Annotated sequences were extracted and InterProScan (5.73–104.0) was used to predict domains, motifs, and functional annotations associated within the *D. reticulatus* genome using inbuilt databases (*E*-value < 1E − 30) [42]. To gain a more comprehensive understanding of the domains and their differences between groups, we searched for the abundance of each Pfam ID within a set of pre-defined groups. These included (1) the *Dermacentor* genus, (2) other aforementioned tick species, (3) blood-feeding insects (*Aedes aegypti:* GCF_002204515.2, *Anopheles gambiae:* GCF_943734735.2, and *Aedes albopictus*: GCF_035046485.1), and (4) non-blood-feeding arachnids (*Centruroides sculpturatus*: GCF_000671375.1 and *Parasteatoda tepidariorum*: GCF_043381705.1) [12, 43–46]. We first calculated the number of each Pfam domain ID and calculated the fold change of *Dermacentor* and all other groups. If the median fold change was > 2, the domain ID was mapped to corresponding GO terms. The median fold change was subsequently estimated for each GO term and input into REVIGO for enhanced visualisation and interpretation [47]. Domains of interest associated with key functions for tick biology were identified and predicted *D. reticulatus* amino acid sequences were identified (*E*-value < 1E − 10). The *D. reticulatus* amino acid sequences were also blasted against the *Dermanyssus gallinae* transcriptome to predict the presence or absence of proteins involved in the heme biosynthesis pathway [48].

### Mitochondria assembly and annotation

The mitochondrial genome was assembled separately from the nuclear genome. Firstly, mitochondrial amino acid sequences were extracted from the *D. silvarum* (GCA_013339745.2) assembly and used to make a DIAMOND v2.1.11 database [49]. The high-quality filtered *D. reticulatus* reads were blasted against this database and filtered to obtain reads that significantly matched the mitochondria reference (*E*-value < 1e^−5^). The remaining reads were sorted and deduplicated using seqtk before undergoing unguided scaffolding using Flye v2.9.2 (–genome-size 15k) [50]. This yielded a single, circular contig which underwent annotation using MitoZ v3.6 [51].

### Ixodida mitochondria tree analysis

All *Dermacentor* mitochondria sequences were downloaded from the NCBI Organelle database (https://www.ncbi.nlm.nih.gov/datasets/organelle/, accessed: March 2026) and the COX1 protein sequences were aligned alongside the *D. reticulatus* mitochondria COX1 protein sequence using MAFFT v7.525 [52]. Sequences included YP_008999737.1 (*Dermacentor nitens*), YP_009122927.1 (*Dermacentor silvarum*), YP_009183446.1 (*Dermacentor nuttalli*), YP_009652696.1 (*Dermacentor everestianus*), YP_010231340.1 (*Dermacentor auratus*), YP_010283870.1 (*Dermacentor andersoni*), YP_010286580.1 (*Dermacentor variabilis*), YP_010324938.1 (*Dermacentor steini*), YP_010324951.1 (*Dermacentor marginatus*), YP_010324964.1 (*Dermacentor niveus*), YP_010329480.1 (*Dermacentor sinicus*), YP_010509210.1 (*Dermacentor albipictus*), and YP_010571307.1 (*Dermacentor reticulatus*) [53–65]. A maximum-likelihood tree was built using IQ-TREE v 2.3.6 with 1000 bootstraps and automated model finder selection (-B 1000 -m MFP -T AUTO). The mitochondria tree was visualised using custom scripts.

## Results

### Assessment of genome assembly quality and statistics

In total, after quality filtering we retained 20,972,661 reads totalling 89.24 Gb, with a mean read length of 4255 bp, median length of 2952 bp, and N50 of 5795 bp, corresponding to an estimated genome coverage of 36.6 × based on an assumed genome size of 2.44 Gb. K-mer frequency analysis was performed using Jellyfish and modelled with GenomeScope 2.0 (k = 27), estimating a haploid genome size of 2,128,482,012 bp, a heterozygosity rate of 1.34%, and a sequencing error rate of 0.441%, with the model accounting for 98.7% of the observed k-mer space (Supplementary Fig. S1). The moderate heterozygosity is consistent with values reported for other tick species and was accounted for by assembling with Hifiasm, which employs a haplotype-resolved approach to minimise haplotype duplication in the primary assembly [23, 24]. Overall, ONT sequencing yielded good quality unguided and guided *D. reticulatus* assemblies that are comparable to those for other *Dermacentor* species (Table 1). The guided assembly resulted in 1248 scaffolds, which was intermediate compared to other *Dermacentor* species: fewer than *D. silvarum* (1665 scaffolds) but more than *D. andersoni* (791 scaffolds) and *D. albipictus* (568 scaffolds). The use of *D. silvarum* to guide scaffolding increased the number of scaffolds and facilitated the formation of putative chromosomes, leading to a greater scaffold N50 of 216 MB. *D. silvarum* was selected because it is the most closely related species to *D. reticulatus*, supported by phylogenomic analysis of mitochondrial sequences and has a publicly available high-quality, chromosome-level genome assembly [61]. A total of 11 putative chromosomes were formed, matching the chromosome numbers reported for *D. silvarum* and *D. andersoni*, and aligning with previous karyotyping studies of *Dermacentor* species [26]. However, only 10 chromosomes are reported for *D. albipictus*, possibly reflecting either a genuine biological difference or methodological variation in assembly.

**Table 1:** Quality assessment and BUSCO scores (arachnida_odb10) of unguided and guided *Dermacentor reticulatus* assemblies in comparison to other *Dermacentor* sp.

Assembly statistics	*Dermacentor silvarum*^a^	*Dermacentor andersoni*^b^	*Dermacentor albipictus*^c^	*Dermacentor reticulatus* (unguided)	*Dermacentor reticulatus* (guided)
Number of scaffolds	1665	791	568	–	1248
Number of contigs	15,182	836	679	3595	2890
Percent gaps	0.09%	0.04%	0.02%	< 0.01%	< 0.01%
Scaffold N50	189 MB	205 MB	181 MB	–	216 MB
Contigs N50	339 KB	66 MB	30 MB	2 MB	3 MB
Putative number of chromosomes	11	11	10	–	11
Genome size	2.5 GB	2.4 GB	2.2 GB	2.4 GB	2.4 GB
GC content	47%	46.5%	46.5%	46.5%	46.5%
BUSCO score	96.8%	97.0%	97.1%	97.9%	97.9%
Complete BUSCOs (C)	2841	2847	2849	2873	2873
Complete and single-copy BUSCOs (S)	2687	2782	2742	2766	2766
Complete and duplicated BUSCOs (D)	154	65	107	107	107
Fragmented BUSCOs (F)	42	31	35	14	14
Missing BUSCOs (M)	51	56	50	47	47

^a^*D. silvarum* (GCA_013339745.2), ^b^*D. andersoni* (GCA_023375885.3), and ^c^*D. albipictus* (GCA_038994185.2)

The 11 putative chromosomes in the *D. reticulatus* genome ranged in size from 118 to 535 Mb, with chromosome 1 being the largest. Chromosomes were labelled by descending size, following standard convention. GC content was also comparable across genome assemblies for all species (~ 46.5%). Combined, this demonstrates the utility of ONT sequence for rapid assembly of tick species with adequate quality, as demonstrated by previous studies [66].

**Fig. 1:**
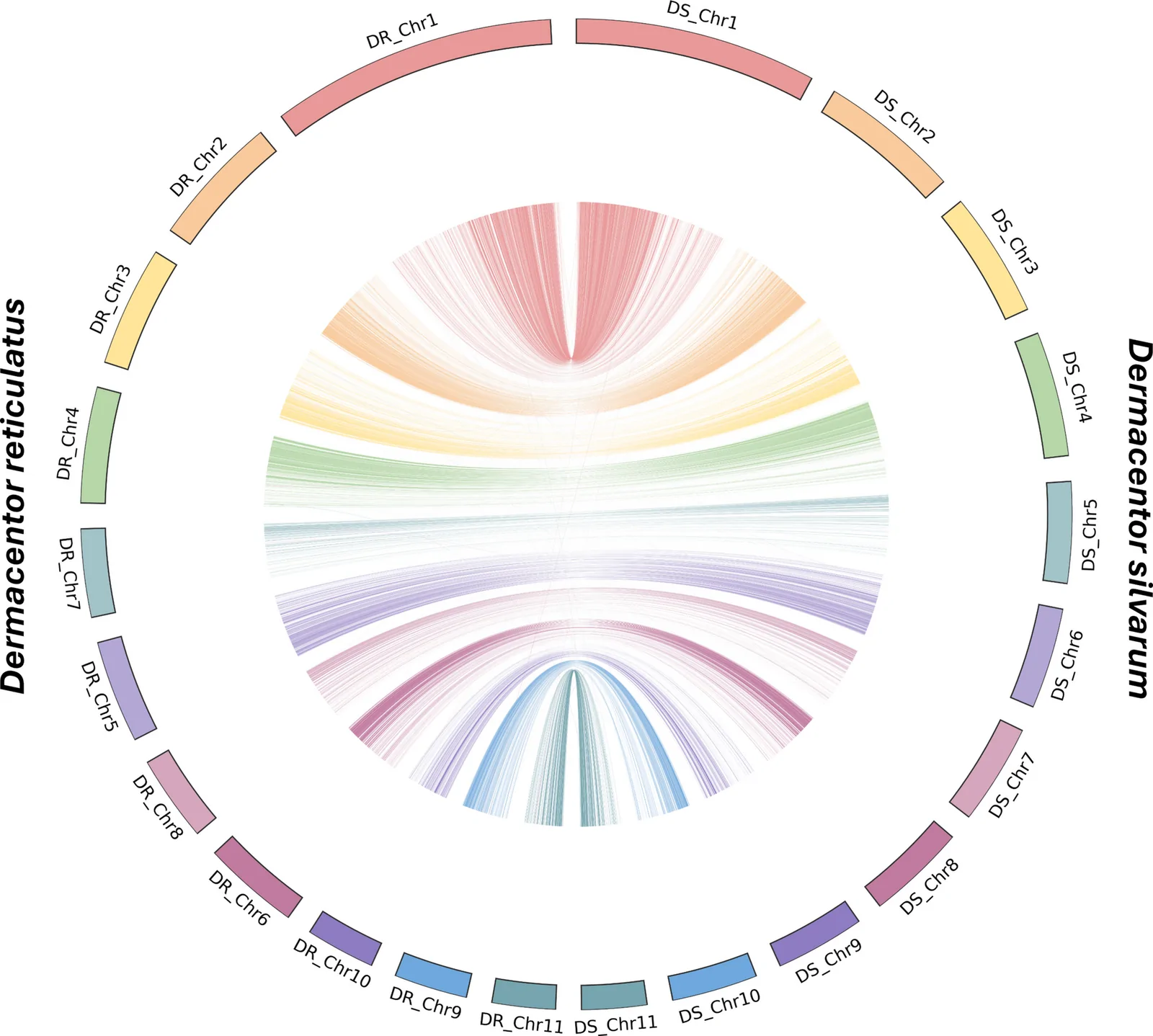
Nucleotide-level synteny of *Dermacentor reticulatus* and *Dermacentor silvarum* genomes. Nucleotide-level synteny between *Dermacentor reticulatus* (DR) and *Dermacentor silvarum* (DS) is shown for the repeat-masked guided *D. reticulatus* assembly. Scaffolds and links are coloured according to corresponding *D. silvarum* chromosomes

The BUSCO (Benchmarking Universal Single-Copy Orthologs) scores of *D. reticulatus* assemblies (unguided and guided: 97.9%) were comparable to *D. silvarum* (96.8%), *D. andersoni* (97.0%), and *D. albipictus* (97.1%). The number of complete and single-copy genes was also high across all *Dermacentor* assemblies (> 2800), representing the completeness of each genome and the quality of assembly. The lower number of duplicated, fragmented, or missing BUSCOs suggests the genomes are well-assembled and are free from misassemblies, duplications, or gaps within these regions. Any potential misassemblies were also identified using nucleotide-level synteny visualisation (Fig. 1). The repeat-masked guided assembly demonstrated strong synteny with the *D. silvarum* genome.

### Quality of genome annotation and gene synteny

Prior to annotation, the *D. reticulatus* genome underwent repeat modelling and masking to characterise repetitive elements within the genome. The *D. reticulatus* assembly comprises approximately 63.6% of repeats (Supplementary Fig. S2). Retroelements are the most abundant class, making up 31.30%, and include LINEs (12.25%), LTR elements (18.37%), and SINEs (0.68%). Among LINEs, the R1/LOA/Jockey (5.37%) and L2/CR1/Rex (2.84%) subfamilies are particularly prominent. Within LTR elements, Gypsy/DIRS1 elements dominate at 15.43%, indicating a substantial contribution from ancient retroviral-like sequences. DNA transposons account for 2.95%, with Tc1-IS630-Pogo and hobo-Activator being the most common families. Rolling-circle elements contribute a smaller proportion (0.32%). Notably, a large portion of the genome (26.19%) remains unclassified, highlighting potential novel or highly diverged repeat families yet to be characterised. Additional minor components include simple repeats (2.08%), small RNAs (0.66%), satellites (0.06%), and low-complexity regions (0.07%). This composition reflects the repetitive nature of the *D. reticulatus* genome and its evolutionary history, with a predominance of retrotransposon activity and a high degree of uncharacterised repetitive sequence content.

**Fig. 2:**
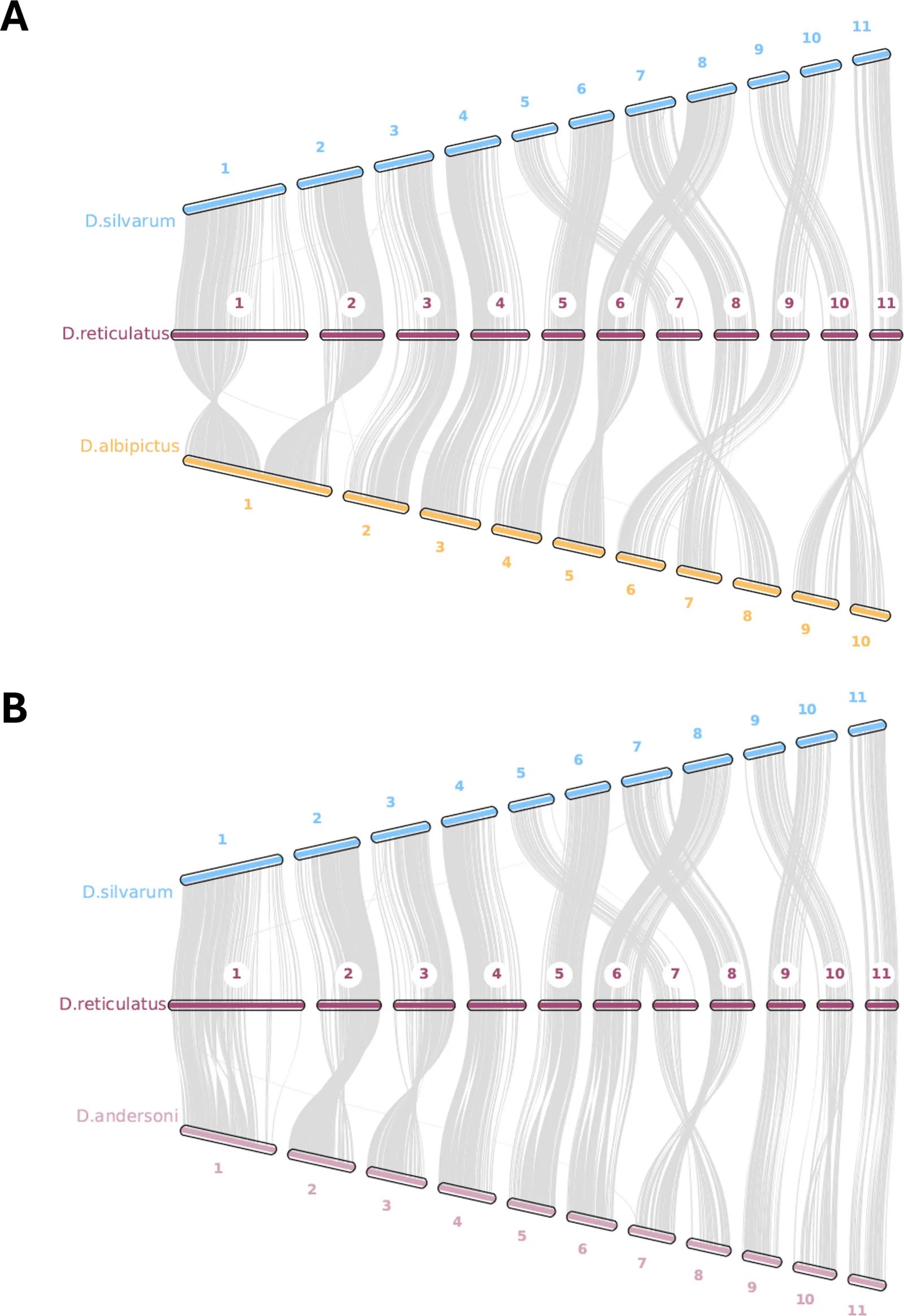
Gene-level synteny between the guided *Dermacentor reticulatus* assembly and members of the *Dermacentor* genus. Gene-level synteny (1:1 orthologous regions) between the guided *D. reticulatus* genome assembly and *D. silvarum* (guiding reference; GCA_013339745.2 [12, 67]). Comparisons are also made to *D. albipictus* (GCA_038994185.2) (**A**) and *D. andersoni* (GCA_023375885.3) (**B**). Only synteny between (putative) chromosomes are shown. Blocks are coloured according to each species

Annotation quality was assessed using BUSCO and compared against other *Dermacentor* assemblies (Supplementary Table S1). The *D. reticulatus* assemblies yielded BUSCO completeness scores of 98.0%, comparable to those of *D. silvarum*, *D. andersoni*, and *D. albipictus*, all with approximately 98%. The unguided assembly did not increase the quality of annotation, suggesting minimal impact of scaffolding on gene model recovery. Furthermore, we also compared gene synteny across the *Dermacentor* assemblies (Fig. 2). Notably, *D. reticulatus*, *D. silvarum*, and *D. andersoni* all have 11 chromosomes, while only 10 are reported for *D. albipictus*. Gene order across the *Dermacentor* assemblies was largely conserved, with chromosomal rearrangements observed between species. In *D. albipictus*, chromosome 1 appears to represent a fusion of chromosomes 1 and 2 as defined in *D. reticulatus* and *D. silvarum*, consistent with the reduced chromosome number. A minor intrachromosomal inversion was also observed on chromosome 4 of *D. albipictus*. Comparison with *D. andersoni* revealed several intrachromosomal inversions across chromosomes 2, 3, 4, 5, 10, and 11, suggesting greater structural rearrangement relative to *D. reticulatus* than observed in *D. silvarum* or *D. albipictus*. However, it should be noted *D. silvarum* was used to guide the formation of chromosomes for *D. reticulatus*, which is reflected in its high synteny. For chromosomes 2, 3, and 7 of *D. andersoni*, whole-chromosome orientation differences are apparent, likely reflecting differences in assembly strategy or Hi-C scaffolding interpretation rather than true biological inversions. Despite these structural differences, gene order along chromosomes was well-conserved across all species (Fig. 2).

### Mitochondrial genome analysis of tick species

**Fig. 3:**
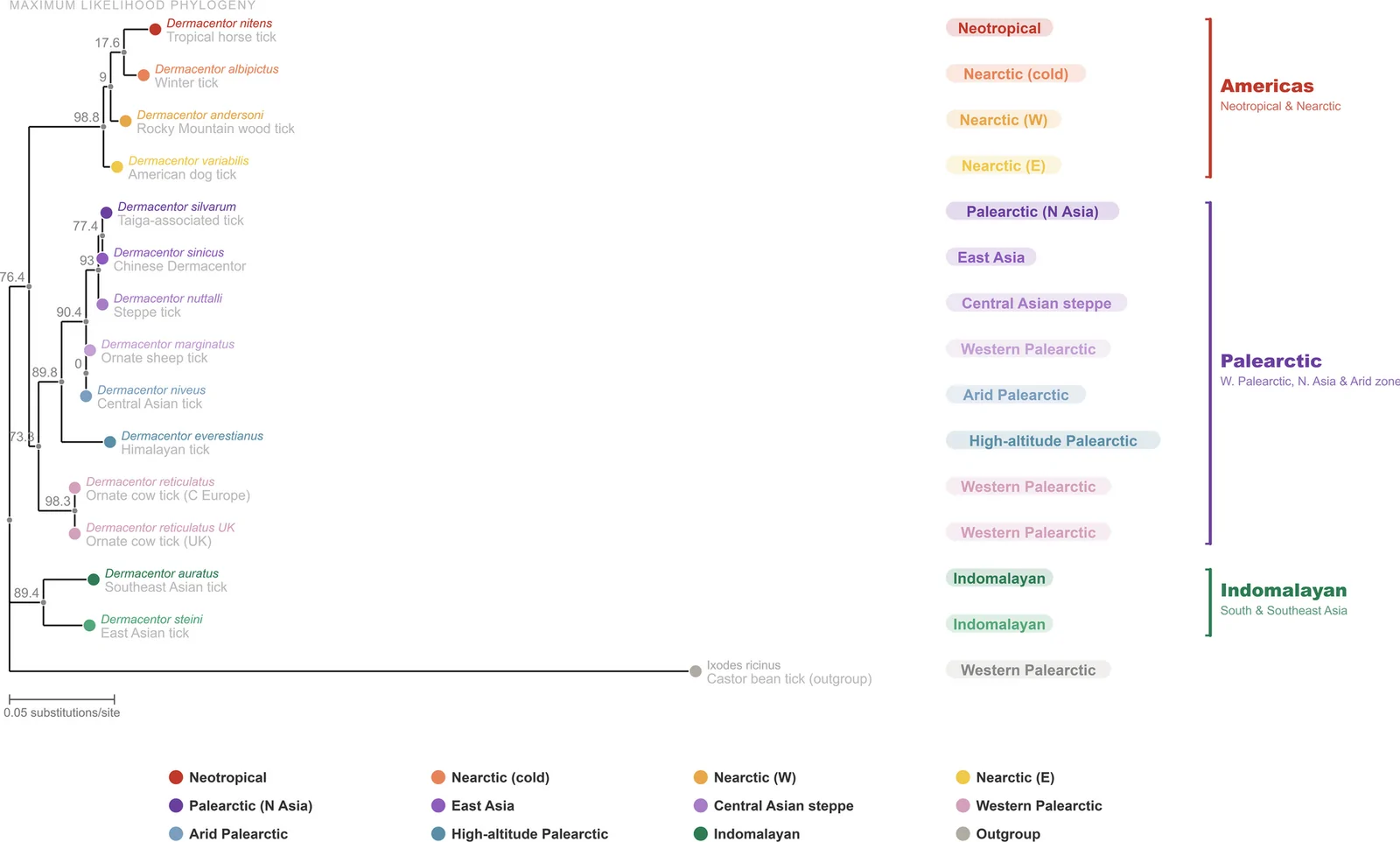
Maximum-likelihood tree of *Dermacentor* mitochondria COX1 protein sequences. A maximum-likelihood tree was constructed using an alignment of 15 mitochondria COX1 protein sequences across the *Dermacentor* genus and *Ixodes ricinus* outgroup. The new reference for *D. reticulatus* is highlighted (UK), showing expected clustering within the *Dermacentor* genus and an existing *D. reticulatus* COX1 sequence (YP_010571307.1). Tips are coloured and labelled according to tick geographic distribution. Bootstrap supports are shown on branches

We also assembled and annotated the mitochondrial genome of *D. reticulatus*, which is 15,103 bp in length and contains 38 genes, including 23 tRNAs and 2 rRNAs (Supplementary Fig. S3). A comparison to 13 publicly available *Dermacentor* mitochondrial genomes and *Ixodes ricinus* outgroup (COX1 gene), including a previously published *D. reticulatus* mitogenome (NC_068757.1 [61]), confirmed the completeness and accuracy of our assembly, with strong clustering of the two *D. reticulatus* sequences (Fig. 3).

Phylogenetic analysis of mitochondrial COX1 sequences revealed broader evolutionary relationships across the *Dermacentor* genus. Within *Dermacentor*, phylogenetic patterns largely reflected geographic distribution. *D. reticulatus* clustered among *D. silvarum*, *D. marginatus*, *D. nuttalli*, *D. sinicus*, *D. niveus*, and *D. everestianus*, suggesting a shared evolutionary history across Europe, Central, and East Asia. *D. auratus* and *D. steini*, found in South and Southeast Asia, also share a close relationship. North American species such as *D. albipictus*, *D. andersoni*, and *D. variabilis* are less closely related to *D. reticulatus*, consistent with their distinct geographic origins.

### Comparison of gene orthologues of tick species

**Fig. 4:**
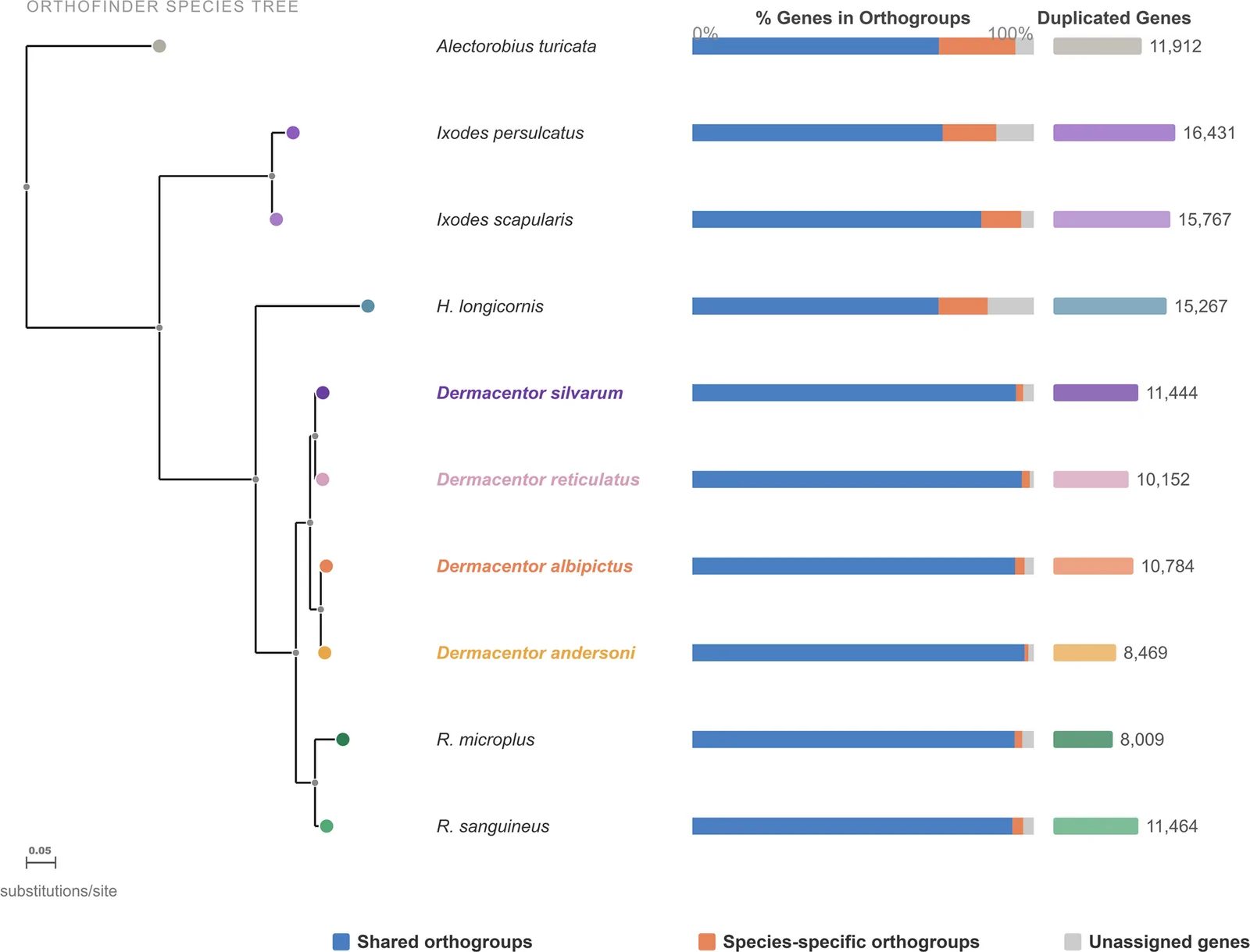
Comparative analysis of gene orthologues in tick species. Maximum-likelihood trees constructed using gene orthologues and duplications across tick species, including *D. albipictus* (GCF_038994185.2), *D. andersoni* (GCA_023375885.3), *D. silvarum* (GCF_013339745.2), *Haemaphysalis longicornis* (GCA_013339765.2), *Ixodes scapularis* (GCF_016920785.2), *Ixodes persulcatus* (GCA_013358835.2), *Alectorobius turicata* (GCF_037126465.1), *Rhipicephalus microplus* (GCF_013339725.1), and *Rhipicephalus sanguineus* (GCF_013339695.2) [12, 15, 16, 67, 68]. Trees are rooted on the *A. turicata* outgroup. Additional comparative genomic statistics are also shown. The first column of bars indicates the percentage of genes belonging to non-species-specific orthogroups, species-specific orthogroups, and unassigned genes. The second column of bars indicates the number of gene duplications and is labelled with the corresponding number of duplications

We further performed a comparison between annotated genome sequences to gain a comprehensive overview of gene evolution, orthology, and duplications (Fig. 4, Supplementary Table S2). The guided *D. reticulatus* genome assembly contained 21,592 predicted genes, similar to the gene counts observed in other tick species. A total of 98.8% of *D. reticulatus* genes were assigned to orthogroups. A maximum-likelihood phylogenetic tree built from single-copy orthologous genes supported expected evolutionary relationships, clustering *Dermacentor* species more closely with other members of the *Ixodidae* family, while *Alectorobius turicata* (a soft tick from the family *Argasidae*) formed an outgroup (Fig. 4). Different patterns were observed for gene duplication events. A total of 10,152 gene duplication events were observed for *D. reticulatus*, similar to values for *D. albipictus* and *D. silvarum* (Fig. 4).

### Comparative functional genomics and GO term analysis

Following the analysis of tick evolutionary relationships based on orthologous and mitochondrial sequences, additional insight into key biological adaptations and traits underlying tick evolution was gained through protein family (Pfam) domain comparisons and gene ontology analysis (Supplementary Tables 3–11). These approaches have previously been applied in genomic studies of other tick species and provide a broader functional context for interpreting evolutionary changes [12, 34]. In the absence of biological replicates, we compared the fold change of each domain and its associated GO term between members of the *Dermacentor* genus and three other groups: (1) other tick species, (2) blood-feeding insects [BFI: *Aedes aegypti*, *Anopheles gambiae*, and *Aedes albopictus*], and (3) non-blood-feeding arachnids [NBFA: *Centruroides sculpturatus* and *Parasteatoda tepidariorum*] (see “Methods”), similar to Jia et al. [12]. Across all comparisons, the majority of enriched GO terms corresponded to molecular function (other ticks: 60; BFI: 302; NBFA: 178), followed by biological processes (other ticks: 64; BFI: 265; NBFA: 174) and cellular components (other ticks: 27; BFI: 135; NBFA: 78). These enriched terms covered a wide range of functions, many of which may reflect evolutionary adaptations relevant to hematophagy and vector competence (Supplementary Tables S3–S11).

Among the most highly enriched GO terms across comparisons were those related to lipid biology, including lipid transport (GO:0006869), lipoprotein metabolic processes (GO:0042157), and lipid binding (GO:0008289), each showing a fold change of 54.27 relative to other tick species. Amine binding (GO:0043176) showed the highest enrichment compared to both BFIs and NBFAs (fold change: 4077.44 in both), alongside terms related to symbiont-mediated perturbation of host defences (GO:0030682; fold change: 2061.31 vs both BFI and NBFA). Notable enrichment of cysteine-type endopeptidase inhibitor activity (GO:0004869; fold change: 421.02) and RNA-directed RNA polymerase activity (GO:0003968; fold change: 354.88) was also observed when comparing *Dermacentor* to BFIs. Compared to NBFAs, defence response (GO:0006952; fold change: 277.69), protein N-linked glycosylation (GO:0006487; fold change: 186.30), and beta-1,4-mannosylglycoprotein 4-beta-N-acetylglucosaminyltransferase activity (GO:0003830; fold change: 186.30) were among the most enriched terms, the latter consistent with the previously reported enrichment of mannosyl-glycoprotein-related functions in *Dermacentor*. Several enriched GO categories across all comparisons were also related to broader nutrient metabolism, including lipid, carbohydrate, glucose, fatty acid, amino acid, mannose, and fructose metabolism, as well as cellular components such as the extracellular matrix (Supplementary Tables S3–S11).

Cholesterol metabolism was enriched in *Dermacentor* relative to both BFIs (GO:0008203, fold change: 8.04; GO:0006694, fold change: 4.53) and NBFAs (GO:0008203, fold change: 3.44; GO:0006694, fold change: 2.56), but was absent from the tick comparison, consistent with ticks sharing a dependence on host-derived cholesterol. Ticks and mosquitoes cannot synthesise cholesterol de novo and must acquire it from external sources. However, mosquitoes can convert plant-derived sterols into cholesterol via modification pathways, whereas ticks, being obligate blood feeders, depend entirely on their vertebrate hosts for cholesterol acquisition [69]. The enrichment of cholesterol metabolism-related GO terms in *Dermacentor* may reflect adaptations to this dependency. Enrichment of metalloendopeptidase activity (GO:0004222; fold change: 7.09) and proteolytic activity more broadly (GO:0006508; fold change: 10.81) was also observed when comparing *Dermacentor* to NBFAs, suggesting capacity for extracellular matrix degradation relevant to host tissue penetration during attachment and feeding.

Among the protease inhibitors, we detected 33 proteins with the core cystatin domain (IPR000010) and 78 proteins containing the core serpin domain (IPR023796), indicating active roles for these inhibitors in modulating host immune responses and regulating proteolytic activity during blood feeding (Supplementary Table S12). Within the defensin family, 18 hits were found for the invertebrate/fungal defensin domain (IPR001542), suggesting a meaningful antimicrobial peptide repertoire in this species (Supplementary Table S12). For detoxification-related proteins, we identified 704 cytochrome P450 domain-containing proteins (IPR001128), 114 carboxylesterases (IPR002018), and multiple glutathione S-transferases (94 hits for the N-terminal domain [IPR004045] and 41 for the C-terminal domain [IPR004046]). Additionally, 411 proteins contained ABC transporter-like domains (IPR003439) and 158 had ABC transporter transmembrane domains (IPR011527), suggesting a substantial expansion of ABC transporter-mediated efflux mechanisms in *D. reticulatus* (Supplementary Table S12). These domain profiles support the presence of complex and specialised molecular systems in *D. reticulatus* for managing oxidative stress, host interactions, and xenobiotic detoxification [12, 70–73].

To investigate the heme biosynthesis pathway in *D. reticulatus*, we searched for homologs of key enzymes using *Dermanyssus gallinae* proteins as references. Homologs for the late pathway enzymes, including protoporphyrinogen oxidase (PPOX) and ferrochelatase (FECH), were identified (1.31E − 133 and ≤ 8.09E − 136, respectively), indicating conservation of the terminal steps of heme biosynthesis (Supplementary Table S13). However, no matches were detected for several early enzymes in the pathway, such as 5-aminolevulinate synthase (ALAS1/ALAS2), ALA dehydratase (ALAD), porphobilinogen deaminase (HMBS), uroporphyrinogen III synthase (UROS), and uroporphyrinogen decarboxylase (UROD), suggesting these genes may be absent. This finding aligns with previous reports indicating that Metastriata ticks have lost enzymes involved in the early stages of the pathway [34].

## Discussion

*Dermacentor reticulatus* is a carrier of several tick-borne pathogens, including *Babesia*, *Borrelia*, *Anaplasma*, *Rickettsia*, *Bartonella*, *Francisella*, *Coxiella*, and tick-borne encephalitis virus [6]. Due to anthropogenic and climate factors, *D. reticulatus* has expanded its range to regions previously uninhabited by this tick species [3]. Consequently, effective surveillance of *D. reticulatus* is crucial for managing the risk of transmitting tick-borne diseases that pose substantial medical and veterinary threats. Such surveillance schemes include the UKHSA Tick Surveillance Scheme (TSS) that has been implemented in the UK [4, 74]. Since 2024, *D. reticulatus* has been reported using TSS in known endemic locations (e.g. Wales, Essex, and South West England) [5]. While some of the ecology and behaviour of *D. reticulatus* is better understood, its genome remains largely unexplored and no chromosome-scale genome assembly is currently available. Genomes of other tick species, such as *Ixodes scapularis* (the black-legged tick), have been sequenced and annotated in detail [12, 41]. There remains a limited understanding of the unique molecular and biological adaptations in *D. reticulatus*, which might differ significantly from other ticks. Therefore, a high-quality reference genome for *D. reticulatus* will provide a foundation for downstream comparative genomic analysis, enabling more detailed comparisons with other tick species and potentially revealing novel genes or pathways that could be targeted for tick surveillance or disease prevention.

We have shown the genome assemblies of *D. reticulatus* are of comparable quality to existing reference genomes within the *Dermacentor* genus. We included both unguided and guided assemblies, along with their annotations, to capture the structural accuracy and improved contiguity provided by the guided assembly, while also acknowledging that reference-based guidance may introduce bias [75]. Our analysis reveals that, despite differences in chromosome orientation and potentially chromosome number across the *Dermacentor* genus, there is conservation of nucleotide sequences and gene synteny among these species. Subtle differences may be observed due to differences in the assembly approach used. For example, unlike previous studies, we utilised long-read ONT data to generate the draft assembly. Incorporating Hi-C and Illumina data could have further improved the assembly by enhancing chromosomal-level scaffolding and correcting potential structural variations [12]. However, we demonstrate the utility of ONT data for rapid, cost-effective genome assembly of a non-model organism using high molecular weight DNA, especially for complex organisms, including ticks. The use of ONT data offers significant advantages for assembling large, repetitive genomes like that of *D. reticulatus*, which we estimate to be approximately 63.6% repetitive [76]. As a result, we have generated both unguided and guided assemblies of *D. reticulatus*, each approximately 2.4 Gb in size, achieving high BUSCO scores (> 97%), indicating strong completeness and accuracy in the genome reconstruction. However, we acknowledge several limitations of the current assembly. The proportion of unclassified repetitive elements remains high, reflecting the poor representation of *Dermacentor*-specific repeats in current databases, and future work would benefit from de novo repeat library construction to improve annotation completeness. Additionally, the use of reference-guided scaffolding, while improving contiguity, may have introduced biases in regions where the *D. reticulatus* genome diverges substantially from the reference. Nevertheless, this assembly represents the first high-quality genome for *D. reticulatus*, providing a valuable resource for future studies into tick biology, vector competence, and the development of novel control strategies.

We further annotated the *D. reticulatus* genome assembly to predict genes. We performed an orthologous gene analysis to explore the evolutionary relationships and functional similarities of genes in *D. reticulatus* compared to other tick species, including *D. albipictus*, *D. andersoni*, *D. silvarum*, *H. longicornis*, *I. scapularis*, *I. persulcatus*, *O. turicata*, *R. microplus*, and *R. sanguineus*. Our analysis involved identifying orthologous genes across these species, providing insights into their shared ancestry and functional conservation. This work identified a significant number of orthologous gene clusters across all species, suggesting that while ticks diverged along different evolutionary paths, many genes have been conserved throughout the Ixodida order. These conserved genes are likely to play fundamental roles in basic cellular processes, such as metabolism, DNA repair, and immune response, which are critical for tick survival and adaptation to various environmental niches and hosts [12, 34]. We also observed differences in the number of duplicated genes between *D. reticulatus* and other members of the *Dermacentor* genus, which may have contributed to species diversification.

Our functional domain and GO enrichment analyses shed light on several molecular adaptations in *D. reticulatus* that support its hematophagous lifestyle [12]. The identification of detoxification-related domains, including cytochrome P450s, carboxylesterases, glutathione S-transferases, and ABC transporters, suggests a capacity to process host-derived and environmental xenobiotics, as well as to mitigate oxidative stress associated with blood feeding [70, 71]. Similarly, the presence of abundant serpin and cystatin domains highlights tight protease regulation, likely crucial for controlling host responses and maintaining gut homeostasis during prolonged feeding [77]. The presence of 18 invertebrate/fungal defensin domain-containing proteins (IPR001542) suggests an active antimicrobial peptide repertoire in *D. reticulatus*, potentially contributing to pathogen control during blood feeding [72, 73].

Importantly, we observed strong enrichment of metalloendopeptidase-related GO terms in *Dermacentor* compared to non-blood-feeding arachnids. These enzymes, often involved in extracellular matrix degradation, may facilitate host tissue penetration or remodelling during tick attachment and feeding. For example, thiol-activated metalloendopeptidase with kininase activity, a protease enzyme that breaks down peptides, has been observed in *Boophilus microplus* saliva (cattle tick) [78]. The enrichment of cholesterol metabolism-related GO terms in *Dermacentor* was also of interest. As obligate blood feeders, ticks cannot synthesise cholesterol and must acquire it entirely from their vertebrate hosts. In contrast, mosquitoes retain the ability to modify plant sterols, reducing their dependence on host cholesterol [79]. The overrepresentation of cholesterol-associated pathways in *D. reticulatus* may therefore represent enhanced systems for uptake, trafficking, or utilisation of host-derived lipids. Further, while late stage heme biosynthetic enzymes (PPOX and FECH) were present in *D. reticulatus*, earlier pathway components were not detected, consistent with the hypothesis that Metastriata ticks have lost the ability to synthesise heme. This suggests a reliance on host-derived heme, as previously observed for *D. silvarum* and other tick genera [12, 34].

Finally, we present the mitochondrial genome of *D. reticulatus*, along with a phylogenetic tree constructed from mitochondrial data (COX1 protein sequences) available across *Dermacentor*. The mitochondrial genome has a rapid evolutionary rate, is maternally inherited, and has a relatively conserved structure across species. As such, mitochondrial markers are important for inferring evolutionary relationships, especially among closely related species within a genus or across genera [56]. The mitochondrial maximum-likelihood tree we constructed clarifies the evolutionary position of *D. reticulatus* within the *Dermacentor* genus and reveals notable geographic patterns, suggesting the influence of geographic barriers or migration routes on its distribution. Given the high conservation of the mitochondrial genome in *D. reticulatus*, additional nuclear genomic markers will be essential for identifying signatures of geographical adaptation within this species.

## Conclusion

In summary, we have generated the first draft reference genome for *D. reticulatus* using ONT long-read data and provided a mitochondrial sequence alongside a phylogenetic tree, which reveals important geographic patterns within the genus. The annotated genome offers insights into key genes and biological processes, establishing a valuable resource for future population genetic studies and for advancing our understanding of tick evolution, one-health, and infection control.

## Supplementary Information

Supplementary Material 1.

Supplementary Material 2.

## Data Availability

All raw Oxford Nanopore Technology sequencing reads and genome assemblies generated in this study have been deposited at NCBI under BioProject accession PRJNA1418530 (BioSample: SAMN55046655). The whole genome shotgun assembly has been deposited at DDBJ/ENA/GenBank under accession JBXYWG000000000; the version described in this paper is JBXYWG010000000 [31]. Data are also available through the LSHTM Genomics Data Repository (https://genomics.lshtm.ac.uk/data/) [32].

## Abbreviations

ONT: Oxford Nanopore Technologies
DR: *Dermacentor reticulatus*
DS: *Dermacentor silvarum*
BUSCO: Benchmarking Universal Single-Copy Orthologs
BFI: Blood-feeding insects
NBFA: Non-blood-feeding arachnids
