# Supplementary material for: The first chromosome-scale genome assembly for Dermacentor reticulatus: a key vector of tick-borne pathogens of public and veterinary health importance in Europe
Source: BMC Biol. 2026 Jun 11;24:185. doi: 10.1186/s12915-026-02646-z (PMC13495156; doi:10.1186/s12915-026-02646-z)
Supplement: Supplementary file 1 [file 12915_2026_2646_MOESM1_ESM.docx]

**Supplementary Figures**

**Supplementary Figure S1. GenomeScope 2.0 k-mer frequency profile of the tick genome.** K-mer histogram generated from Jellyfish (k = 27) shown on a linear (left) and log (right) scale. Dashed vertical lines indicate k-mer coverage peaks. The model estimates a haploid genome size of ~2.13 Gb, heterozygosity of 1.34%, and sequencing error rate of 0.441%, accounting for 98.7% of the observed k-mer space.

**Supplementary Figure S2. Repeat content of the *Dermacentor reticulatus* genome visualised as a Sankey diagram.**
 The figure illustrates the hierarchical classification and relative abundance of repetitive elements identified in the *D. reticulatus* genome assembly. The diagram shows the flow from total repeat content (63.6% of the genome) through major repeat classes to their constituent subfamilies.

**Supplementary Figure S3. Mitochondria Sequence and Annotation for *D. reticulatus.*** The circular mitochondria sequence for *D. reticulatus* is shown alongside its annotation and genome coverage.
